# BMI trajectories, morbidity, and mortality in England: a two‐step approach to estimating consequences of changes in BMI

**DOI:** 10.1002/oby.23510

**Published:** 2022-08-03

**Authors:** Laura A. Gray, Penny R. Breeze, Elizabeth A. Williams

**Affiliations:** ^1^ Health Economics and Decision Science, School of Health and Related Research University of Sheffield Sheffield UK; ^2^ Healthy Lifespan Institute University of Sheffield Sheffield UK; ^3^ Department of Oncology and Metabolism University of Sheffield Sheffield UK

## Abstract

**Objective:**

BMI is known to have an association with morbidities and mortality. Many studies have argued that identifying health risks using single BMI measures has limitations, particularly in older adults, and that changes in BMI can help to identify risks. This study identifies distinct BMI trajectories and their association with the risks of a range of morbidities and mortality.

**Methods:**

The English Longitudinal Study of Aging provides data on BMI, mortality, and morbidities between 1998 and 2015, sampled from adults over 50 years of age. This study uses a growth‐mixture model and discrete‐time survival analysis, combined using a two‐step approach, which is novel in this setting, to the authors' knowledge.

**Results:**

This study identified four trajectories: “stable overweight,” “elevated BMI,” “increasing BMI,” and “decreasing BMI.” No differences in mortality, cancer, or stroke risk were found between these trajectories. BMI trajectories were significantly associated with the risks of diabetes, asthma, arthritis, and heart problems.

**Conclusions:**

These results emphasize the importance of looking at change in BMI alongside most recent BMI; BMI trajectories should be considered where possible when assessing health risks. The results suggest that established BMI thresholds should not be used in isolation to identify health risks, particularly in older adults.

**Study Importance**

**What is already known?**

- BMI has been shown to be associated with mortality and multiple morbidities.
- Changes in BMI as well as current BMI have been shown to be important predictors of health outcomes.

**What does this study add?**

- Results from this study show that heterogeneity in BMI trajectories is important and can explain significant differences in health outcomes.
- BMI trajectory has little association with all‐cause mortality but has a significant and substantial association with diabetes.

**How might these results change the direction of research or the focus of clinical practice?**

- It is standard for cost‐effectiveness analyses to assume a single mean trajectory, minimizing heterogeneity. Results from this study could be used to better inform cost‐effectiveness analysis or economic evaluation.
- The findings could help primary care professionals to earlier identify individuals who are at increased risk of negative health outcomes following changes to their BMI as they get older.

## INTRODUCTION

BMI is known to have an association with morbidity and mortality. Being underweight or living with obesity has been associated with higher risk of all‐cause mortality compared with having a “healthy” BMI [1]. In adults, having a higher BMI has been linked with a higher chance of diabetes [2, 3], hypertension [4], arthritis [5], depression and anxiety [6, 7], cardiovascular disease [8], stroke [9], heart disease [2, 3], cancers [10], and mortality [11].

Despite BMI being a well‐established measure of adiposity in adults, many studies have previously argued that defining health risks using the current BMI thresholds is inappropriate, particularly in older adults [12, 13, 14]. In particular, there has been a lack of consensus of the health risks associated with having overweight, with some studies suggesting a reduced risk of mortality compared with a healthy BMI [15, 16, 17]. This is known as the overweight risk paradox. Research has suggested that this may be due to a failure of BMI to account for the distribution of body fat [18] or confounders such as smoking [3], rather than a protective effect of having overweight. Current guidance from the National Institute for Health and Care Excellence (NICE) suggests that established BMI thresholds should not be used in isolation; further research is needed into appropriate ways to identify health risks as individuals get older. Nevertheless, the established BMI thresholds continue to be used to assess health risks.

Changes in BMI, as well as thresholds measured at a single time point, could be appropriate for identifying health risks [19, 20]. Using a single measure of BMI cannot identify individuals who spend time with high levels of BMI but later lose weight or vice versa. Research has investigated changes in BMI and its association with a range of outcomes. Increased risk of mortality has been associated with increasing BMI [20, 21, 22] as well as decreasing BMI [20, 23]. Diabetes‐related mortality has been associated with decreasing BMI [23], although this could possibly be due to reverse causality. Increasing BMI has been associated with higher risks of various cancers [24] and with stroke and heart disease [25]. Dementia has also been linked with living with obesity during midlife [26] and decreasing BMI [26, 27]. Links between BMI trajectories in childhood and asthma have been extensively researched [28, 29, 30], but less so in adults.

The studies mentioned here have looked at individual health outcomes. We investigated the effects of changes in BMI on mortality and multiple morbidities in older adults living in England. First, we identified distinct BMI trajectories in adults over the age of 50 years. Next, we determined whether these distinct BMI trajectories translate into differing risks of multiple morbidities and mortality, simultaneously. We aim to inform public health policy makers and to more thoroughly identify individuals at increased risk of obesity‐related mortality and morbidities who may be overlooked when using a single measure of BMI alone.

## METHODS

### Data

The English Longitudinal Study of Aging (ELSA) recruited 18,813 participants from the 1998 to 2000 samples of the Health Survey for England (HSE) who were 50 years or older on March 1, 2002. This longitudinal study uses data from the HSE (referred to from this point as wave 0) and waves 1 to 7 of the ELSA questionnaires. At waves 2, 4, and 6 of the ELSA, nurse visits were offered to eligible participants (online Supporting Information Appendix B). During these visits, height and weight were measured by a nurse, and the resulting BMI (kilograms per meters squared) was calculated. There were approximately 4 years between each of these nurse visits and, including wave 0, data on BMI are available from 1998 to 2013.

Data were linked with National Health Service (NHS) central register official mortality data, providing accurate mortality data, available from waves 1 to 6 (2012). Data on whether the participant had suffered from morbidities (type 2 diabetes, cancer, arthritis, asthma, heart problems, stroke) were self‐reported during every wave from wave 1 to wave 7 (2014). We used self‐reported morbidities because of a large number of missing values on doctor‐diagnosed variables.

Baseline characteristics associated with BMI trajectory included participant's age (in years), sex (male/female), ethnicity (White/non‐White), smoking status (smoker/nonsmoker), and marital status (married or cohabiting/unmarried) and were chosen in reference to existing studies [15, 20]. More descriptive ethnic groups were not possible because of the very high proportion (>97%) of White participants. Sex was included because male individuals have been shown to have a lower baseline BMI [20, 23, 31]. Both male and female individuals were included in the same analysis because previous studies have found that there is little difference between estimated BMI trajectories of male and female individuals [20].

This study used a structural equation modeling framework to link a growth‐mixture model (GMM) with a discrete‐time survival analysis (DTSA). A visual path diagram showing both parts of the full structural equation modeling can be found in online Supporting Information Appendix A.

### GMM

GMMs [32] have been used to estimate multiple distinct BMI or body‐shape trajectories in older adults [20, 21, 24, 33]. GMMs have advantages over other longitudinal models, for example, random coefficient models [34], in that they allow the identification of distinct subpopulations with similar trajectories within a heterogeneous population [35]. GMMs use a multinomial modeling approach to determine whether heterogeneity in the population is a consequence of a finite number of distinct groups or, simply, that all individuals are different.

We used the baseline characteristics outlined previously to estimate baseline BMI and the probability of following each distinct BMI trajectory. Aggregated data showed a quadratic relationship between age and BMI; therefore, intercept, slope, and quadratic terms were included to model BMI over time, similar to previous studies [23, 24, 31, 36], and we compared models with one to five latent trajectories. The optimal number of latent components is determined by comparing Bayesian information criterion (BIC), entropy, the mean probabilities of each component, and the intuitiveness of the resulting trajectories in accordance with the literature [20, 23, 32, 37]. Further details on the GMM estimation can be found in online Supporting Information Appendix A.

### DTSA

We investigated associations between health risks and distinct BMI trajectories using DTSA [38]. We assumed proportional odds of the hazards of each event (i.e., an individual had the same likelihood of death between each wave; online Supporting Information Appendix A). Data were censored independently for each DTSA after death or diagnosis; this did not affect the censoring in other outcomes.

The residual variance of each survival model was unrestricted, creating a random effect allowing for unobserved heterogeneity in the propensity to experience each event. Hazard ratios were adjusted for age, sex, ethnicity, smoking, and marital status. Further details on the DTSA estimation can be found in online Supporting Information Appendix A.

### Bolck, Croon, and Hagenaars two‐step approach

The Bolck, Croon, and Hagenaars (BCH) approach [38, 39] uses a weighted ANOVA, with weights inversely related to the classification error probabilities; it does not explicitly assign individuals to a single trajectory and minimizes the potential impact of reverse causality by preventing outcomes from explicitly influencing trajectories. This means that, if a person is diagnosed with a morbidity, they remain in the estimated BMI trajectories. However, if someone dies, they would no longer be included in the data and thereby would not contribute to the BMI trajectories or the morbidity DTSA.

The BCH approach has been shown in other settings to substantially outperform other approaches [40]. To the best of our knowledge, this is the first study to use a two‐step approach to investigate changes in BMI with multiple DTSAs. Step 1 estimates the GMM (BMI trajectories) independent of the outcomes of interest (health risks), preventing distal outcomes (such as subsequent mortality) from influencing prior BMI trajectories. Step 2 is the estimation of the outcomes, in this case, DTSA, estimating the risks of morbidities and mortality using BMI trajectory as a predictor. Step 2 does not influence step 1 and, therefore, gets around problems associated with joint estimation and allows simultaneous estimation of multiple outcomes.

Missing data were assumed to be missing at random. More details on missing data and the patterns of missing data can be found in online Supporting Information Appendix B. Missing BMI values did not result in observations being removed from the analysis as long as at least one BMI value was available. The vast majority of observations (97.1%) had at least two BMI values during the period of study.

Data manipulation was performed in Stata version 17 (StataCorp LLC), and analysis was carried out in Mplus version 8.7 (Muthén & Muthén).

## RESULTS

Because of the availability of baseline characteristics and data from at least one nurse visit, 9,206 individuals over the age of 50 years were included in our analysis. Around 85% of those eligible participated in each of the nurse visits [41]. Table 1 shows the sample means, standard deviations (SD), and number of observations for variables of interest. Table 1 also shows the proportion of participants who died since the previous wave and the proportion of individuals who newly reported (since the previous wave) having each of the morbidities. Mean observed BMI was consistently in the overweight range (25 ≤ BMI < 30), and it increased gradually over time. Very few participants had underweight (BMI < 18.5) in any wave.

**FIGURE 1:**
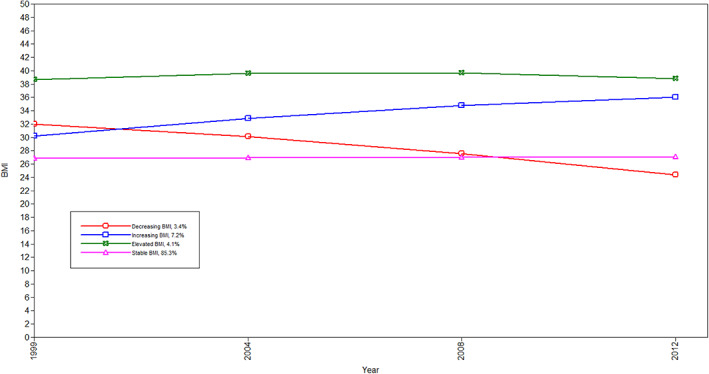
BMI trajectories estimated using GMM. GMM, growth‐mixture model [Color figure can be viewed at wileyonlinelibrary.com]

Approximately 46% of the sample were male, and 98% were White. At baseline, just under 18% identified as being smokers, declining significantly over the subsequent waves to under 9% by wave 7.

### BMI trajectories

**FIGURE 2:**
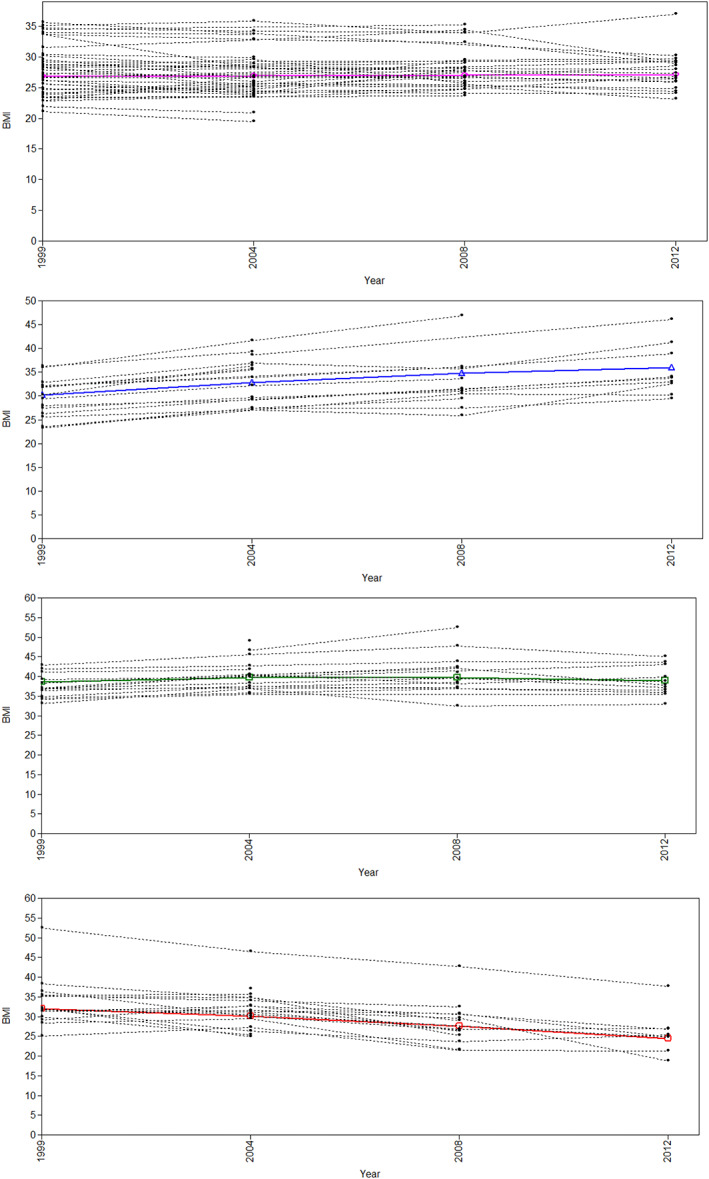
Variation within estimated trajectories [Color figure can be viewed at wileyonlinelibrary.com]

We identified four distinct BMI trajectories, shown in Figure 1: “stable overweight” (mean BMI consistently around 27); “elevated BMI” (mean BMI consistently around 40); “increasing BMI” (mean BMI around 30 at baseline and increasing to 37 throughout the study period); and “decreasing BMI” (mean BMI around 32 at baseline and reducing to around 25). Each trajectory represents an unobserved subpopulation, and each individual has a posterior probability of following each trajectory. Figure 2 shows each estimated trajectory and a random sample of observed trajectories to visualize the distribution of variation around each trajectory. The estimated trajectories fit the observed data well, and there is a good degree of separation between them.

**TABLE 1:** Characteristics of HSE and ELSA participants in the study sample

	Wave 0 (1998‐2000)	Wave 1	Wave 2	Wave 3	Wave 4	Wave 5	Wave 6	Wave 7
BMI, (kg/m^2^), mean (SD)^a^	27.69 (4.537)	—	27.88 (4.813)	—	28.19 (5.102)	—	28.21 (5.047)	—
	*n* = 8,549		*n* = 6,485		*n* = 6,594		*n* = 5,484	
People with underweight (BMI ≤ 18), n (%)	35 (0.4)	—	23 (0.4)	—	20 (0.3)	—	10 (0.2)	—
People with normal weight (18 < BMI ≤ 25), n (%)	2,394 (28.0)	—	1,721 (26.5)	—	1,740 (26.4)	—	1,465 (26.7)	—
People with overweight (25 < BMI ≤ 30), n (%)	3,912 (45.8)	—	2,812 (43.4)	—	2,843 (43.1)	—	2,368 (43.2)	—
People with obesity (BMI > 30), n (%)	2,208 (25.8)	—	1,929 (29.7)	—	1,991 (30.2)	—	1,641 (29.9)	—
Age (y), mean (SD)^a^	61.72 (8.635)	—	—	—	—	—	—	—
	*n* = 9,206							
Male (%)^a^	45.74	—	—	—	—	—	—	—
	*n* = 9,206							
White (%)^a^	97.76	—	—	—	—	—	—	—
	*n* = 9,206							
Married (%)^a^	70.24	70.56	68.40	67.18	68.1	67.2	65.8	64.8
	*n* = 9,206	*n* = 7,150	*n* = 6,901	*n* = 6,258	*n* = 7,529	*n* = 6,952	*n* = 6,456	*n* = 5,625
Smoker (%)^a^	17.5	16.2	13.9	12.6	12.3	11.1	9.9	8.8
	*n* = 9,206	*n* = 7,128	*n* = 6,899	*n* = 6,256	*n* = 7,452	*n* = 6,898	*n* = 6,458	*n* = 5,625
Mortality (%)^a^	—	—	—	2.0	3.1	4.1	3.7	—
				*n* = 8,967	*n* = 8,785	*n* = 8,510	*n* = 8,161	
Diabetes (%)^a^ ^,^ ^b^	—	2.1	1.3	1.4	1.6	2.3	1.7	2.4
		*n* = 8,802	*n* = 8,618	*n* = 7,888	*n* = 7,234	*n* = 6,513	*n* = 5,851	*n* = 4,952
Cancer (%)^a^ ^,^ ^b^	—	1.5	1.5	1.2	1.3	2.7	2.1	2.9
		*n* = 8,792	*n* = 8,658	*n* = 7,927	*n* = 7,284	*n* = 6,598	*n* = 5,910	*n* = 4,961
Arthritis (%)^a^ ^,^ ^b^	—	7.2	5.9	4.2	4.4	5.7	6.0	5.4
		*n* = 6,947	*n* = 6,447	*n* = 5,663	*n* = 5,063	*n* = 4,439	*n* = 3,867	*n* = 3,147
Asthma (%)^a^ ^,^ ^b^	—	2.0	1.1	0.5	0.9	0.7	0.6	0.6
		*n* = 8,349	*n* = 8,186	*n* = 7,514	*n* = 6,926	*n* = 6,262	*n* = 5,677	*n* = 4,825
Heart problems (%)^a^ ^,^ ^b^	—	3.0	2.7	1.7	3.1	4.4	4.3	5.7
		*n* = 8,166	*n* = 7,917	*n* = 7,217	*n* = 6,676	*n* = 5,960	*n* = 5,286	*n* = 4,374
Stroke^a^ ^,^ ^b^	—	0.5	0.8	0.6	1.0	1.0	1.4	1.4
		*n* = 8,976	*n* = 8,934	*n* = 8,234	*n* = 7,611	*n* = 6,895	*n* = 6,273	*n* = 5,328

*Notes*: This table displays the characteristics of the 9,206 participants in the sample used in the analysis in this study. Mortality data linked to ELSA from the National Health Service central register was available only to wave 6.

Abbreviations: ELSA, English Longitudinal Study of Aging; HSE, Health Survey for England.

^a^ *n* = number of observations with nonmissing BMI values.

^b^ Data are percentage with new diagnosis since previous wave.

A four‐component model was determined to be an improvement on three components when comparing the BIC and entropy, and it also gave the most intuitive results. Adding a fifth trajectory increased the fit of the model according to the BIC, but the probability of following the additional trajectory was negligibly small (0.3%) and, therefore, it added little to interpretation. Details on model selection can be found in online Supporting Information Appendix C. Results discussed from this point refer to the four‐component quadratic model.

Table 2 shows the mean probabilities of trajectory membership and the odds ratios (OR) for following each trajectory relative to the stable overweight trajectory, by baseline characteristic. Individuals are most likely (85.3%) to follow the stable overweight trajectory. The mean probability of being in the increasing BMI trajectory is 7.2%; the odds of following this trajectory significantly reduce with age, are significantly lower in male individuals, and are significantly increased for smokers compared with the stable overweight trajectory. The mean probability of following the elevated BMI trajectory is 4.1%; the odds are significantly reduced in older individuals and are significantly lower in male individuals and in married individuals compared with the stable overweight trajectory. The mean probability of following the decreasing BMI trajectory is 3.4%; odds are higher in older individuals and in female individuals and smokers compared with the stable overweight trajectory. Ethnicity has no significant association with the odds of following a particular BMI trajectory.

**TABLE 2:** OR for probability of trajectory membership

	Stable overweight (reference component)	Increasing BMI	Elevated BMI	Decreasing BMI
Mean probability of component membership	85.3%	7.2%	4.1%	3.4%
Age, OR (95% CI)	1	**0.946 (0.928‐0.965)**	**0.973 (0.956‐0.989)**	**1.095 (1.061‐1.130)**
Male, OR (95% CI)	1	**0.442 (0.337‐0.578)**	**0.254 (0.163‐0.396)**	**0.192 (0.105‐0.354)**
White, OR (95% CI)	1	1.234 (0.470‐3.240)	0.492 (0.144‐1.680)	1.440 (0.170‐12.201)
Smoker, OR (95% CI)	1	**2.485 (1.895‐3.260)**	0.750 (0.479‐1.174)	**1.787 (1.077‐2.965)**
Married, OR (95% CI)	1	0.880 (0.690‐1.122)	**0.557 (0.389‐0.797)**	0.714 (0.481‐1.061)

*Note*: English Longitudinal Study of Aging (ELSA) and Health Survey for England (HSE), *N* = 9206. Entropy = 0.868. Bold indicates statistical significance (95% confidence level). Abbreviation: OR, odds ratio.

### Health outcomes

Table 3 shows the hazard ratios for mortality and morbidities for each BMI trajectory relative to the trajectory with the largest probability, stable overweight. We found no association between mortality and BMI trajectory. Similarly, there is no significant association between BMI trajectory and the likelihood of cancer or stroke.

**TABLE 3:** HR for morbidities and mortality

	Stable overweight (reference trajectory)	Increasing BMI	Elevated BMI	Decreasing BMI
Mean probability of trajectory membership	85.3%	7.2%	4.1%	3.4%
Mortality, HR (95% CI)	1	0.756 (0.408‐1.398)	1.028 (0.574‐1.840)	1.129 (0.797‐1.599)
Diabetes, HR (95% CI)	1	**3.687 (2.721‐4.997)**	**5.744 (4.107‐8.034)**	**4.603 (2.393‐7.209)**
Cancer, HR (95% CI)	1	0.873 (0.568‐1.343)	1.308 (0.848‐2.017)	1.080 (0.636‐1.835)
Arthritis, HR (95% CI)	1	**1.697 (1.337‐2.154)**	**1.600 (1.121‐2.283)**	1.132 (0.728‐1.761)
Asthma, HR (95% CI)	1	**1.752 (1.139‐2.694)**	**2.272 (1.367‐3.774)**	**2.557 (1.358‐4.818)**
Stroke, HR (95% CI)	1	1.475 (0.875‐2.487)	0.792 (0.339‐1.852)	1.181 (0.657‐2.124)
Heart problems, HR (95% CI)	1	1.250 (0.932‐1.676)	**1.579 (1.136‐2.194)**	1.236 (0.829‐1.844)

*Notes*: English Longitudinal Study of Aging (ELSA) and Health Survey for England (HSE), *N* = 9206. HR adjusted for baseline characteristics: sex, age, ethnicity, smoking, and marital status. Bold indicates statistical significance (95% confidence level).

Abbreviation: HR, hazard ratio.

There is a significantly increased risk of diabetes in all trajectories compared with the stable overweight trajectory. The risk is highest in the elevated BMI trajectory, in which the risk of developing diabetes is 5.7 (95% CI: 4.1‐8.0) times higher than for the stable overweight trajectory. In the increasing and decreasing BMI groups, this risk is 3.7 (95% CI: 2.7‐5.0) and 4.6 (95% CI: 2.4‐7.2) times higher, respectively. The risk of asthma is also increased in all trajectories when compared with stable overweight; the risk is 1.8 (95% CI: 1.1‐2.7), 2.3 (95% CI: 1.4‐3.8), and 2.6 (95% CI: 1.4‐4.8) times higher in the increasing, elevated, and decreasing BMI trajectories, respectively, compared with the stable overweight trajectory.

Following the increasing or elevated BMI trajectories increases the risk of arthritis by 1.7 (95% CI: 1.3‐2.2) or 1.6 (95% CI: 1.1‐2.3) times, respectively, compared with stable overweight. There is no significant difference in the risk of arthritis between the decreasing BMI and the stable overweight trajectories. The risk of heart problems is 1.6 (95% CI: 1.1‐2.5) times higher in the elevated BMI compared with the stable overweight trajectory, but there is no significant difference in risk for the increasing or decreasing BMI trajectories.

### Secondary analysis

Our results are similar across a number of different subsamples. First, we split the sample by male and female individuals (online Supporting Information Appendix D). In order to explore the influence of existing health on the estimated relationships, we used a subsample of nonsmokers with no long‐standing illness at baseline, referred to as “healthy agers,” in line with previous research (online Supporting Information Appendix E) [18]. We also used complete‐case analysis (online Supporting Information Appendix F) and analysis excluding (BMI < 18) individuals with underweight (online Supporting Information Appendix G). Finally, we adjusted for Indices of Multiple Deprivation quintile to determine whether socioeconomic status plays a role (online Supporting Information Appendix H). Overall, results showed a very similar picture to our main results.

## DISCUSSION

We used the BCH 2‐step approach to simultaneously estimate adjusted hazard ratios for morbidities and mortality associated with four latent BMI trajectories. We predicted multiple health outcomes simultaneously and prevented these outcomes from influencing BMI trajectories as well as overcoming bias caused by classification error.

We identified four latent BMI trajectories: “stable overweight,” “elevated BMI,” “increasing BMI,” and “decreasing BMI.” None of these trajectories lies consistently in the established “healthy BMI” range (18.5 ≤ BMI < 25), which is not unusual in a UK sample of individuals 50 years and above [20, 23]. The most desirable trajectory, in terms of the lowest risk of adverse health, was the stable overweight trajectory. We found that no other trajectory significantly protected against the risk of any of the adverse health outcomes that we investigated compared with the stable overweight trajectory.

The risk of mortality, after adjustment for covariates, did not significantly differ by BMI trajectory, which is in contrast to previous research that has found that both decreasing BMI [20, 23] and increasing [20, 21, 22] BMI were associated with increased mortality. Adjusting for age and other confounders may have led to differences between our results and previous studies that have not done so [20, 23]. This is particularly relevant for decreasing BMI; individuals are more likely to lose weight as they get older as well as having an increased risk of mortality as they age.

Increasing BMI has been associated with higher risk of cancers compared with a lean stable overweight [24]. However, similar to our results, other research has found no relationship between BMI trends and cancer [19]. Further research into different types of cancers that have previously been linked with BMI (colorectal, prostate, breast) [19, 42] could find interesting effects. Furthermore, if a significant number of individuals die with a late diagnosis of cancer, not recorded in the wave prior to death, it is possible that a significant association between BMI trajectory and cancer may have been missed in our analysis.

The risks of diabetes were significantly and substantially increased in all BMI trajectories when compared with the stable overweight trajectory. This suggests that the amount of time individuals spend with a high BMI can contribute to their risk of diabetes, even if they later lose weight; regardless, losing weight appears to dampen the effect when compared with sustaining an elevated BMI. This is consistent with observations from interventions to reduce the incidence of diabetes in high‐risk individuals [43]. Previous studies have also found that decreasing BMI was associated with increased prevalence of diabetes and diabetes‐related mortality [23]. However, it is possible that classification error, given that they assigned individuals to specific trajectory rather than using a probabilistic model, could have influenced their results. This study also split the sample by age groups rather than adjusting for continuous age. We found that increasing BMI was associated with a lower risk of diabetes than decreasing BMI, suggesting that BMI in midlife is important for diabetes risk.

We found that the risk of arthritis was higher in the increasing and elevated BMI trajectories compared with the stable overweight trajectory, supporting previous research that found that an increasing obesity rate in the United States led directly to a larger proportion of arthritis diagnoses [44]. The risks of arthritis were not significantly different in the decreasing BMI trajectory, compared with stable overweight supporting existing research, which suggests that losing weight can help to prevent and treat arthritis [5].

We found an increased risk of asthma for all BMI trajectories compared with the stable overweight trajectory, suggesting that any amount of time spent with BMI over 30 could increase the risk of asthma. Previous studies have found that the risk of asthma was higher in children with an increasing BMI [28, 29] compared with the average, although other research has found that this was the case only in female individuals [30]. We found this relationship was exaggerated in adults but also that the risk of asthma was higher in adults with a decreasing BMI.

We found no association between stroke and BMI trajectory in the participants over age 50 years, despite previous research finding that increases in BMI during adolescence increased the risk of stroke [25]. We found an increased risk of heart problems in the elevated BMI trajectory compared with the stable overweight trajectory, consistent with the literature [25]. However, there was no difference in risk of heart problems between the increasing, decreasing, or stable overweight trajectories.

The external validity of our results depends on the representativeness of our data and its suitability for other generations. We believe the cohort [45] and our sample are representative so as to provide reliable results (online Supporting Information Appendix B). ELSA includes only a small proportion of non‐White participants, meaning that any effect of ethnicity on BMI trajectory may have been missed. We intended to include dementia as one of the morbidities investigated in this study, but this was not possible because data on dementia were unavailable at baseline. Mortality data linked to ELSA from the NHS central register were available only in waves 1 to 6, limiting the analysis on mortality risk. ELSA only captures what happens to individuals from age 50 years and over; therefore, it was not possible to determine what effects earlier BMI has. Further research considering BMI trajectories throughout the entire life course would be of great value and help to understand the effects of BMI in early life on mortality and morbidities, which occur in much greater numbers in later life.

This study is likelihood‐based and assumes data are missing at random, but further research is required into how appropriate this assumption is and whether attrition bias significantly influences these results. It is possible that our data are limited by the inclusion of self‐reported morbidities, used because medical records were not available. Reporting bias or misclassification could exist; for example, individuals' perceived asthma and inhaler use may be associated with other conditions, particularly chronic obstructive pulmonary disease and/or breathlessness. Furthermore, in cases in which patients were diagnosed shortly before death, their diagnosis may not be captured within the data.

Confounding factors might also include other health problems not investigated here, for example, dementia, depression, or reduced self‐care. In order to explore this in more detail, we ran the analysis on a subset of “healthy agers,” individuals who were nonsmokers and did not have any long‐standing illness at baseline. Results were very similar to those reported in the main paper, suggesting that health at baseline did not influence the relationships of interest. However, there may be some health problems that are not considered a long‐term illness, for example, pre‐diabetes; therefore, these individuals could remain in this healthy agers group. These people may receive advice to lose weight as a result of their increased risk of diabetes, meaning that the probability of decreasing BMI could be increased in individuals with worse cardiometabolic health, even after accounting for long‐term conditions. This should be taken into consideration when interpreting our results. Similarly, despite minimizing the impact of reverse causality by using the BCH approach, there could still exist some influence of mortality or morbidities on BMI trajectory, particularly in cases in which the event occurs soon after baseline. For this reason, our study is limited to investigating associations rather than causal influences. Regardless of confounders and causality, recognizing an association between changes in BMI and morbidities or mortality could help speed up the diagnosis of disease by identifying individuals at increased risks.

Further research could help to determine individuals more or less likely to follow each of the identified trajectories. For example, clinical measures and biomarkers of disease (e.g., blood pressure, glycated hemoglobin, cholesterol levels, handgrip strength) could be explored as potential influences. Similarly, life events such as death of a partner or caretaker, divorce, retirement, the birth of a grandchild, or moving into assisted living could influence expected trajectories. Future research could investigate trajectories of waist‐hip ratios and other proxy measures of obesity to determine whether they follow similar patterns over time and lead to similar risks to those we found using BMI.

## CONCLUSION

Despite the stable overweight trajectory being the most desirable trajectory in terms of the health outcomes investigated in this study, the mean BMI in this trajectory is consistently in the overweight category (25 < BMI ≤ 30). The decreasing BMI trajectory has a mean BMI, which is in the normal weight category (18 < BMI ≤ 25), at the most recent measurement but has some significantly worse health outcomes: higher risks of diabetes and asthma. This illustrates the importance of looking at changes in BMI as well as a single BMI measure when identifying risks to health, particularly in an older population.

Results from this study could be used to better inform modeling cost‐effectiveness analysis (CEA) used to assess the value of targeting obesity. It is standard for CEA models to assume an average BMI trajectory, minimizing heterogeneity. However, the results from this study show that heterogeneity is important and that it can explain significant differences in health outcomes. Incorporating heterogeneous trajectories in CEA models would improve the description of the health effects of obesity and capture nonlinearities in the economic benefits of weight gain and weight loss across different groups in the population. Similarly, our findings could be valuable to health care professionals and policy makers. The findings could help primary care professionals to earlier identify individuals who are at increased risk of negative health outcomes after changes to their BMI as they get older.

Our results emphasize the importance of looking at a history of BMI in the patient rather than only the most recent measurement; BMI trajectories should also be considered where possible when assessing health risk. The results cast further doubt on the established thresholds for BMI associated with health risks and suggest that changes in BMI should be used where possible in conjunction with other measures of adiposity, particularly in older adults.

## FUNDING INFORMATION

This study is funded by Medical Research Council (MRC; Grant Number MR/S009868/1) and supported by the National Institute for Health Research (NIHR) School for Public Health Research (SPHR; Grant Number PD‐SPH‐2015). The views expressed are those of the authors and not necessarily those of the MRC, NIHR, or the Department of Health and Social Care.

## CONFLICT OF INTEREST

The authors declared no conflict of interest.

## Supporting information


**Appendix S1** Supporting Information

## Data Availability

The English Longitudinal Study of Aging (ELSA) data sets, including wave 0 from the Health Survey for England (HSE), are available with an End User License at the UK Data Service website (https://beta.ukdataservice.ac.uk/datacatalogue/series/series?id=200011).
