# Supplementary material for: BMI trajectories, morbidity, and mortality in England: a two‐step approach to estimating consequences of changes in BMI
Source: Obesity (Silver Spring). 2022 Aug 3;30(9):1898–907. doi: 10.1002/oby.23510 (PMC9546036; doi:10.1002/oby.23510)
Supplement: Supplementary file 1 — Appendix S1 Supporting Information [file OBY-30-1898-s001.docx]

**Supplementary Material: BMI Trajectories, Morbidity and Mortality in England: A 2-step Approach to Estimating Consequences of Changes in BMI**

**Appendix A: Model Estimation**

The path diagram in Figure A1 uses standard notation, details of which can be found in the Mplus User’s Guide.

*Further details on the Growth Mixture Model (GMM)*

The intercept, slope and quadratic growth factors in the GMM are represented by i ,s and q, respectively. The latent components (i.e. the distinct BMI trajectories) are represented by c. X1 represents independent variables influencing the probability of component membership; age, sex, white, smoker, martial status. X2 represents independent variables used to adjust the hazard ratios for each health outcome; age, sex, white, smoking and martial status.

We estimate model parameters by maximum likelihood using an accelerated expectation maximisation (EMA) algorithm with Fisher Scoring (FS) and Quasi-Newton (QN) optimization as required. We restrict the variance of BMI to be equal at all time points (results are robust when we relax this assumption). Variance of the random intercept (within trajectory variation of baseline BMI) is unrestricted to allow random variation in starting point, reflecting that individuals start with different levels of BMI. We fix the variance of higher order coefficients at zero to avoid overcomplicating the model. All results are robust to different starting values.

*Further details on the Discrete Time Survival Analysis (DTSA)*

Mortality/health indicator variables are represented at each time point by M_t_, D_t_, C_t_, AR_t_, AS_t_, S_t_ and H_t_. These observed indicators are equal to 0 if the patient is alive/undiagnosed, 1 if they died or were diagnoses in period t, and are missing is an individuals died or was diagnosed in a previous period or due to attrition.

Proportional hazards are assumed by fixing the paths to these mortality/health indicator variables to be equal. We attempted to relax this assumption but the model would not converge, probably due to the large increase in parameters that needed estimating. For a more detailed explanation, see Chapter 6 of the Mplus User’s Guide.

Again, we employ maximum likelihood using an EMA algorithm with FS and QN optimization as required.

**Figure A1: Path Diagram**

**
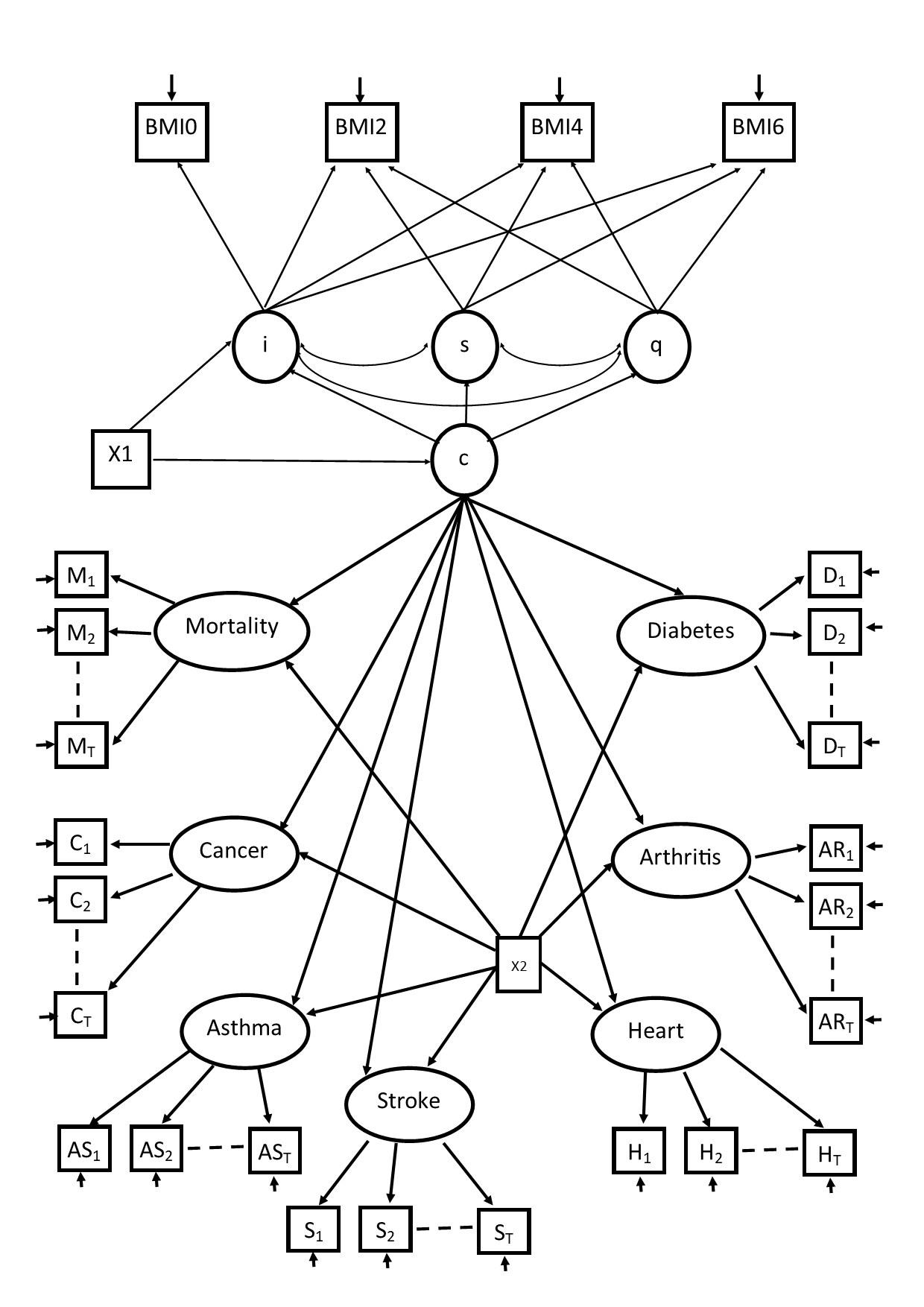
**

**Appendix B: Sample Selection and Missing Data Patterns**

Individuals with at least one ELSA nurse visit during the study period are included in the study sample. Figure B1 shows a flowchart illustrating the number of participants at baseline, those included in the eligibility criteria for ELSA nurse visits and those with valid BMI values at baseline. Not all ELSA participants were eligible for a nurse visit. Nurse visits were offered only to core participants and not their spouses or others in their household, who were also included in the 18,813 participants. For this reason, there is no BMI data on participants which were ineligible for the nurse visits. We believe it is reasonable to assume that those eligible for nurse visits are a reasonably representative sample.

**Figure B1: Flowchart for inclusion/exclusion of participants in wave 0**

Participants at baseline

n=18,813

Meet eligibility criteria with appropriate baseline characteristics

n=9,206

9,607 excluded due to ineligibility for ELSA nurse visits

Meet eligibility criteria for ELSA nurse visit in at least 1 wave

n=12,104

2,898 excluded due missing baseline characteristics

Characteristics of those eligible for the study and those included in the study sample, are available in Table B3 below. As noted in the main text, missing BMI values do not result in observations being removed from the analysis, so long as at least 1 BMI value is available.

Table B1 shows that the vast majority of the eligible sample with sufficient baseline characteristics (97.1%) have non-missing BMI values in at least two of the waves. Almost two-thirds have at least three non-missing waves. Any missing values for BMI do not remove observations from the sample, due to the nature of the GMM analysis. Table B2 shows the different patterns of missingness observed in the sample, along with their frequency.

**Table B1: Frequency in number of missing BMI values**

| **Number missing** | **Frequency** | **Percentage** | **Cumulative %** |
| --- | --- | --- | --- |
| **0** | **2,979** | **32.36%** | **32.36%** |
| **1** | **3,009** | **32.69%** | **65.04%** |
| **2** | **2,951** | **32.06%** | **97.10%** |
| **3** | **267** | **2.90%** | **100.00%** |

**Table B2: Patterns of missing values**

| **Pattern** | **Frequency** |
| --- | --- |
| **++++** | **2,979** |
| **++..** | **1,768** |
| **+.++** | **1,527** |
| **+++.** | **996** |
| **+.+.** | **654** |
| **++.+** | **329** |
| **+..+** | **296** |
| **.+++** | **157** |
| **.+..** | **156** |
| **..++** | **133** |
| **.++.** | **78** |
| **..+.** | **70** |
| **...+** | **41** |
| **.+.+** | **22** |

Table B3 shows the baseline characteristics of participants eligible for nurse visits, those within the study sample and participants by number of missing BMI values. The study sample appear to be reasonably representative of the participants eligible for nurse visits. Age increases with the number of missing values, but this is expected since older participants are more likely to die before the end of the study.

**Table B3: Characteristics by frequency of missing BMI values**

|  |  |  | Number of missing BMI values | | | |
| --- | --- | --- | --- | --- | --- | --- |
|  | Eligible participants | Study sample | 0 | 1 | 2 | 3 |
| N | 12,104 | 9,206 | 2,979 | 3,009 | 2,951 | 267 |
| Age in years  (standard deviation) | 61.72  (8.63) | 61.72  (8.64) | 59.88  (7.31) | 61.41  (8.42) | 63.46  (9.41) | 66.60  (10.03) |
| Male | 45.39% | 45.74% | 49.61% | 46.10% | 47.48% | 45.32% |
| White | 97.03% | 97.76% | 98.46% | 97.47% | 97.36% | 97.75% |
| Married | 70.91% | 70.24% | 74.25% | 68.67% | 68.55% | 61.79% |
| Smoker | 18.66% | 17.5% | 15.11% | 16.48% | 20.98% | 18.35% |

Figure B2 shows a histogram of BMI in the study sample. General population BMI statistics for England are taken from the HSE data which is used in our study. The red line shows all BMI values used in the official statistics, regardless of study eligibility or availability of baseline characteristics. The figure shows that our sample is very representative of the general population in England.

**Figure B2: Baseline BMI in sample vs population**

**Appendix C: Model Selection (GMM)**

The preferred GMM was selected using a range of criteria, including BIC and the intuitiveness of the resulting BMI trajectories. Table C1 shows the BIC, entropy and smallest class probabilities for GMM models with one to five components. BIC decreases as the number of components increased, including form four to five components. However, the addition of an extra component in the five component model, resulted in a BMI trajectory with a mean probability of only 0.3%. Looking at Figures C1 to C3, we can see the BMI trajectories estimated in models with three, four and five components, respectively. This shows that in moving from four to five components, four BMI trajectories remain very similar, with the additional component being the one with very small probability which does not add much to the model interpretation. For this reason, we choose four components as our preferred GMM.

**Table C1: Model Selection**

| # Latent classes | BIC | Entropy | Smallest Class Probability |
| --- | --- | --- | --- |
| 1 | 132338.065 | - | - |
| 2 | 130422.633 | 0.860 | 8.6% |
| 3 | 129424.048 | 0.870 | 3.4% |
| 4 | 128956.498 | 0.868 | 3.4% |
| 5 | 128561.143 | 0.862 | 0.3% |

**Figure C1: BMI Trajectories – 3 Latent Class**


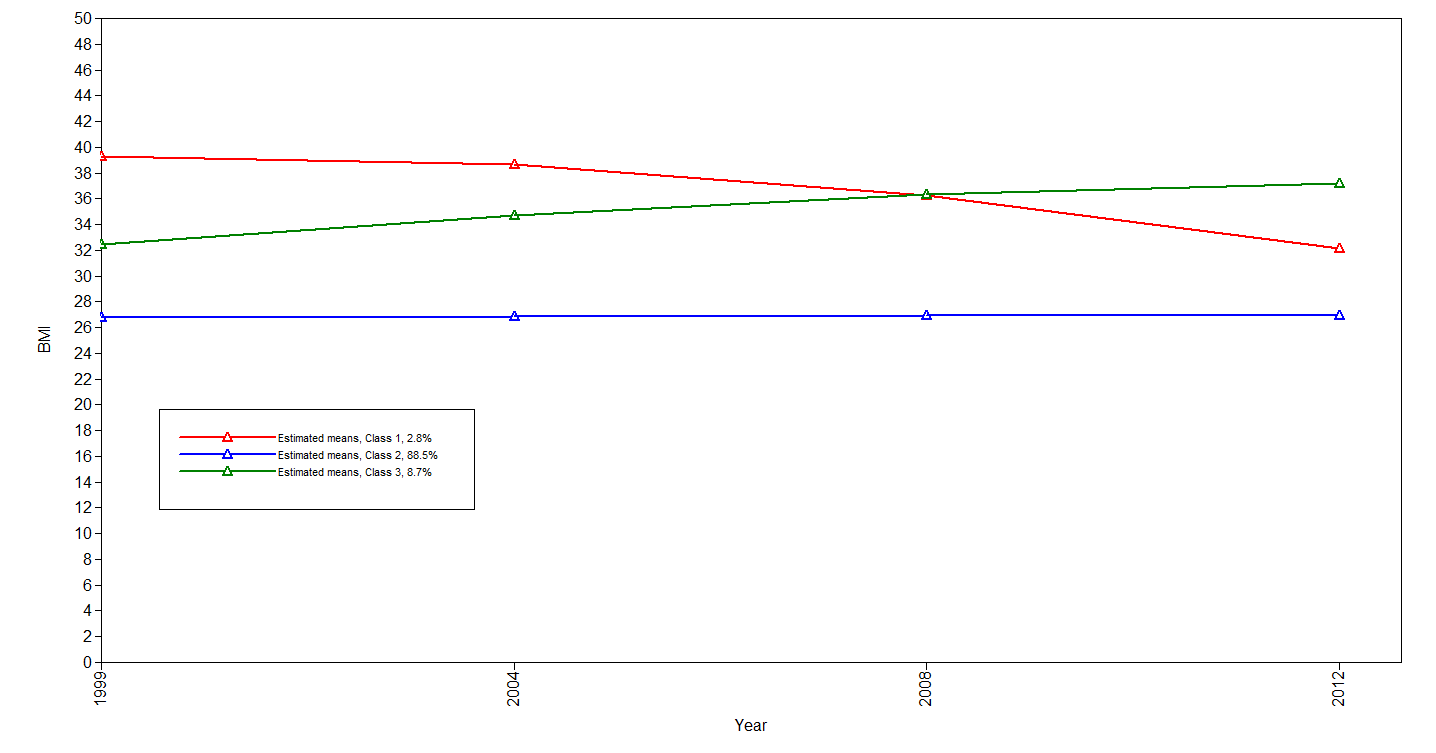


**Figure C2: BMI Trajectories – 4 Latent Class**


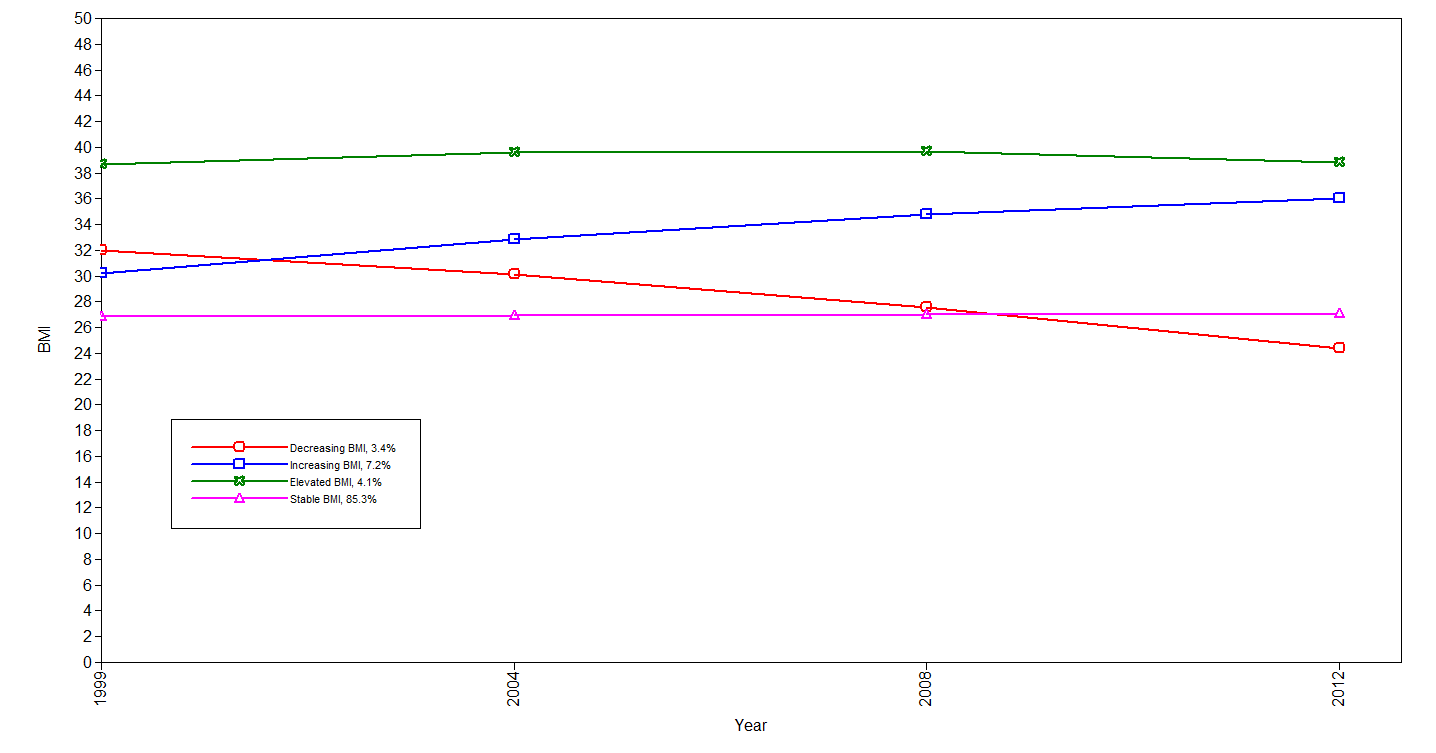


**Figure C3: BMI Trajectories – 5 Latent Class**


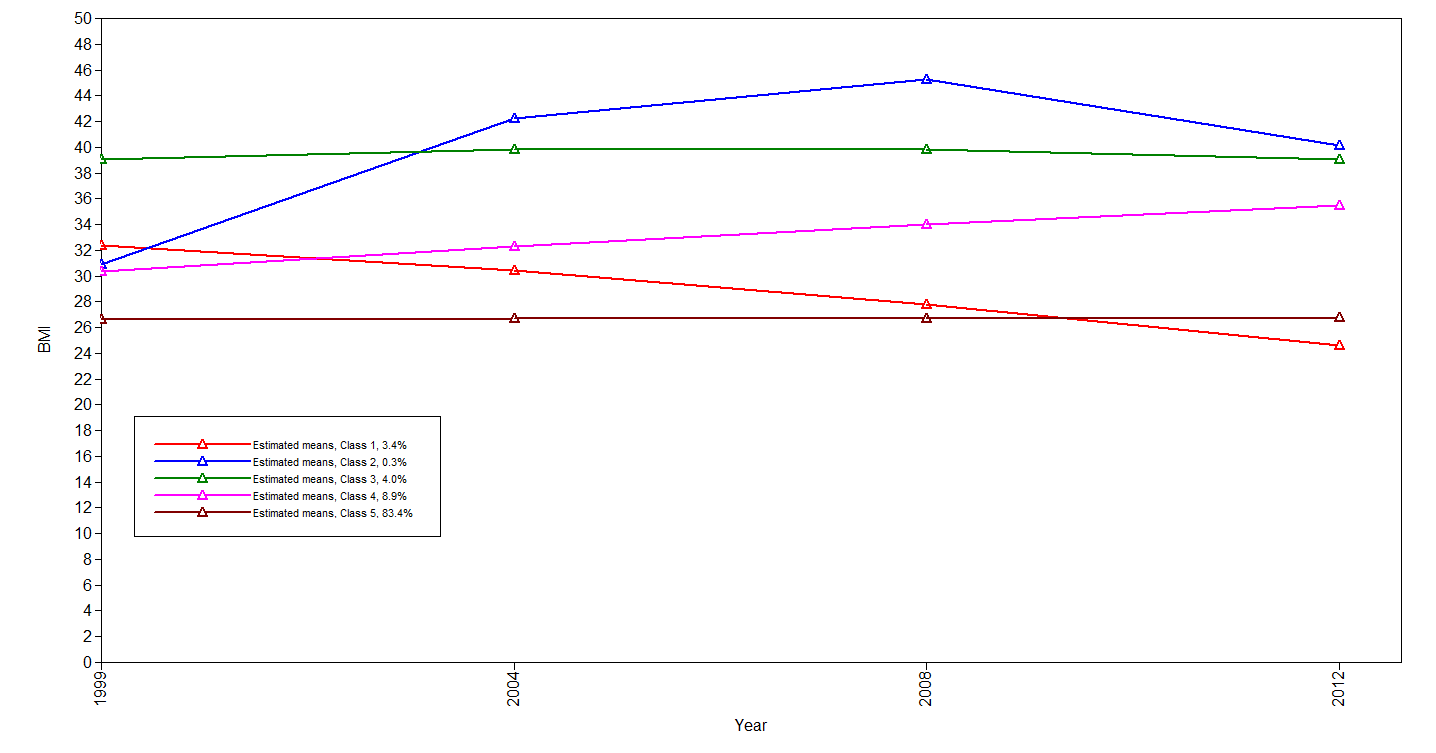


**Appendix D: Analysis for Males and Females**

We repeated the main analysis separately for females and males. Below, are the results for each of these subgroups. Figure D1a and D1b show the distinct trajectories for females and males, respectively. They show the same patterns as the full sample with only minor differences. These tables show that females have a higher probability of following the elevated BMI trajectory, in accordance with the main results (Table 2).

Tables D1a and D1b show the odds ratios for probability of trajectory membership for females and males, respectively. The results look similar to the main results in Table 2. Each of the odds ratios show the effects go in the same direction for males and females. Interestingly, the influence of smoking on the odds of being in the decreasing BMI trajectory, compared to the stable BMI trajectory, is much stronger in males and is insignificant in females. This shows that males were driving this effect in the main analysis. Age is not significant in influencing the odds of being in the elevated or decreasing BMI trajectories compared to the stable BMI trajectory, in males. However, the confidence intervals are very similar in males and females; there is little difference between males and females here.

Tables D2a and D2b show the adjusted hazard ratios for each health outcome in the female and male sample, respectively. Again, the results look similar to the main results in Table 3. All significant hazard ratios show the same direction of effect in both females and males. Diabetes risk is still significantly increase in both males and females following any trajectory other than the stable BMI trajectory, although these effects are stronger in females. Interestingly, the hazard ratios for asthma seem to be driven by females in the increasing and elevated trajectories, but by males in the decreasing trajectory. We find that the hazard ratio for heart problems in the elevated BMI group is insignificant in these subsamples where it was significant in the full sample, possible because of differences in males and females or because of reduced sample size.

**Figure D1a: BMI Trajectories in Females**


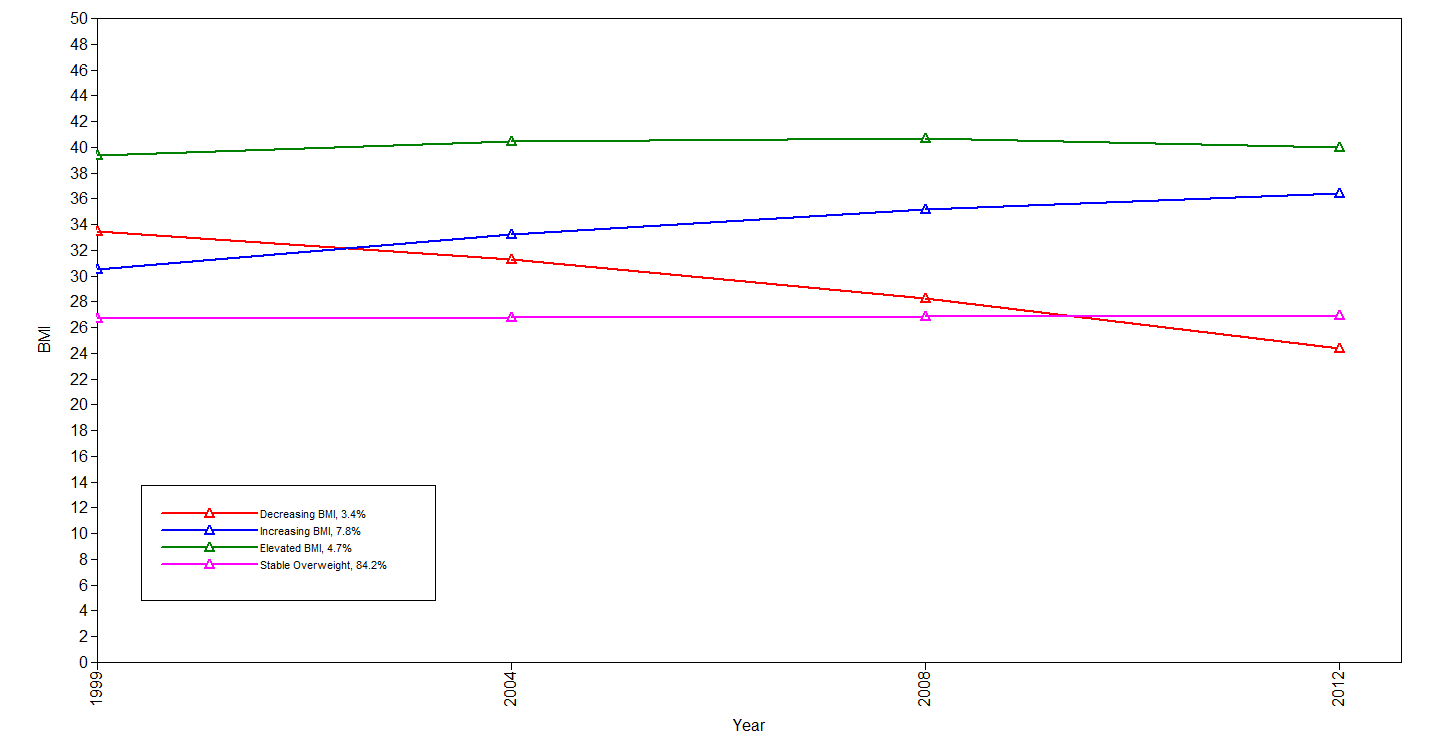
 Data source: English Longitudinal Study of Aging (ELSA) and Health Survey for England (HSE), n=4,995

**Figure D1b: BMI Trajectories in Males**
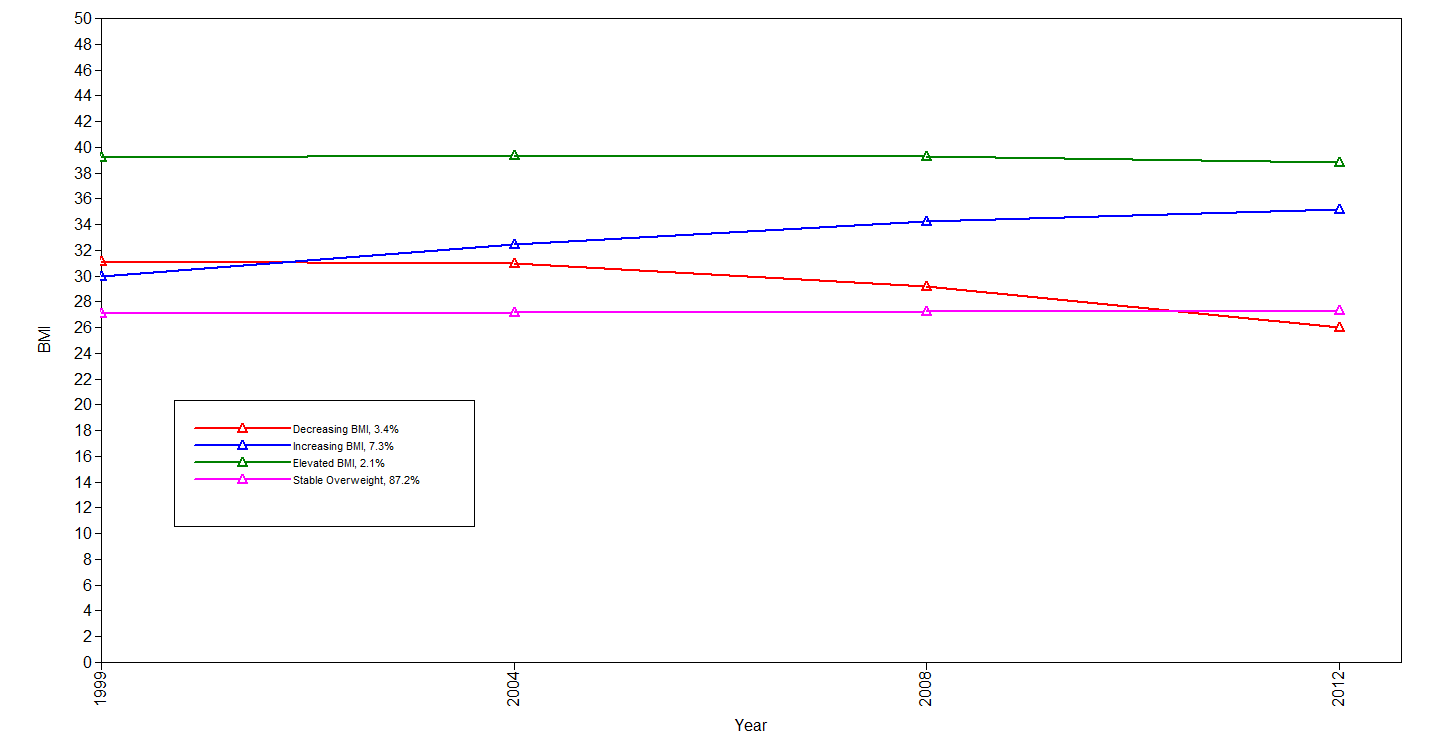


Data source: English Longitudinal Study of Aging (ELSA) and Health Survey for England (HSE), n=4,211

**Table D1a: Odds Ratios for Probability of Trajectory Membership in Females**

|  | **Stable BMI**  **(reference component)** | **Increasing BMI** | **Elevated BMI** | **Decreasing BMI** |
| --- | --- | --- | --- | --- |
| Mean probability of component membership | 84.2% | 7.8% | 4.7% | 3.4% |
| **Odds Ratios for baseline characteristics (95% Confidence Intervals)** | | | | |
| Age (years) | 1 | **0.941**  **(0.914, 0.969)** | **0.967**  **(0.943, 0.991)** | **1.081**  **(1.006, 1.162)** |
| White | 1 | 1.449  (0.260, 8.076) | 1.057  (0.156, 7.180) | 0.729  (0.160, 3.327) |
| Smoker | 1 | **2.299**  **(1.583, 3.341)** | 0.786  (0.456, 1.353) | 1.138  (0.447, 2.899) |
| Married | 1 | 0.896  (0.638, 1.258) | **0.635**  **(0.404, 0.997)** | 0.688  (0.391, 1.210) |

Data source: English Longitudinal Study of Aging (ELSA) and Health Survey for England (HSE), n=4,995. Entropy = 0.852.

**Table D1b: Odds Ratios for Probability of Trajectory Membership in Males**

|  | **Stable BMI**  **(reference component)** | **Increasing BMI** | **Elevated BMI** | **Decreasing BMI** |
| --- | --- | --- | --- | --- |
| Mean probability of component membership | 87.2% | 7.3% | 2.1% | 3.4% |
| **Odds Ratios for baseline characteristics (95% Confidence Intervals)** | | | | |
| Age (years) | 1 | **0.939**  **(0.914, 0.965)** | 0.955  (0.910, 1.002) | 1.083  (0.969, 1.210) |
| White | 1 | 1.151  (0.425, 3.118) | 1.224  (0.128, 11.720) | 0.729  (0.160, 3.327) |
| Smoker | 1 | **2.555**  **(1.726, 3.781)** | 0.314  (0.0.31, 3.186) | **4.088**  **(2.035, 8.215)** |
| Married | 1 | 0.725  (0.472, 1.115) | **0.352**  **(0.180, 0.690)** | 0.497  (0.215, 1.151) |

Data source: English Longitudinal Study of Aging (ELSA) and Health Survey for England (HSE), n=4,211. Entropy = 0.875.

**Table D2a: Hazard Ratios for Mortality and Morbidities in Females**

|  | **Stable BMI**  **(reference trajectory)** | **Increasing BMI** | **Elevated BMI** | **Decreasing BMI** |
| --- | --- | --- | --- | --- |
| Mean probability of trajectory membership | 84.2% | 7.8% | 4.7% | 3.4% |
| **Hazard Ratios (95% Confidence Intervals)** |  |  |  |  |
| Mortality | 1 | 0.777  (0.303, 1.990) | 0.989  (0.435, 2.247) | 1.492  (0.944, 2.297) |
| Diabetes | 1 | **3.327**  **(2.125, 5.209)** | **6.762**  **(4.532, 10.089)** | **3.741**  **(2.159. 6.481)** |
| Cancer | 1 | 1.033  (0.593, 1.801) | 1.413  (0.824, 2.423) | 1.061  (0.509, 2.214) |
| Arthritis | 1 | **1.913**  **(1.391, 2.630)** | **1.657**  **(1.028, 2.671)** | 0.971  (0.537, 1.757) |
| Asthma | 1 | **1.934**  **(1.073, 3.483)** | **2.991**  **(1.711, 5.230)** | 2.073  (0.901, 4.768) |
| Stroke | 1 | 1.781  (0.897, 3.537) | 0.383  (0.058, 2.516) | 1.091  (0.498, 2.388) |
| Heart Problems | 1 | 1.061  (0.687, 1.640) | 1.342  (0.860, 2.094) | 1.246  (0.745, 2.082) |

Data source: English Longitudinal Study of Aging (ELSA) and Health Survey for England (HSE), n=4,995. HRs adjusting for baseline characteristics: age, ethnicity, smoking and marital status.

**Table D2b: Hazard Ratios for Mortality and Morbidities in Males**

|  | **Stable BMI**  **(reference trajectory)** | **Increasing BMI** | **Elevated BMI** | **Decreasing BMI** |
| --- | --- | --- | --- | --- |
| Mean probability of trajectory membership | 87.2% | 7.3% | 2.1% | 3.4% |
| **Hazard Ratios (95% Confidence Intervals)** |  |  |  |  |
| Mortality | 1 | 1.175  (0.645, 2.140) | 1.092  (0.351, 3.394) | 0.379  (0.177, 0.808) |
| Diabetes | 1 | **3.000**  **(2.055, 4.379)** | **4.687**  **(2.617, 8.395)** | **2.550**  **(1.240, 5.244)** |
| Cancer | 1 | 0.726  (0.387, 1.363) | 0.422  (0.071, 2.502) | 1.280  (0.620, 2.643) |
| Arthritis | 1 | **1.758**  **(1.234, 2.503)** | **2.127**  **(1.111, 4.073)** | 1.950  (0.902, 4.217) |
| Asthma | 1 | 1.196  (0.589, 2.428) | 2.373  (0.838, 6.715) | **3.267**  **(1.258, 8.489)** |
| Stroke | 1 | 1.500  (0.713, 3.157) | 0.427  (0.024, 7.469) | 1.076  (0.385, 3.008) |
| Heart Problems | 1 | 1.377  (0.923, 2.053) | 1.719  (0.938, 3.149) | 1.780  (0.938, 3.377) |

Data source: English Longitudinal Study of Aging (ELSA) and Health Survey for England (HSE), n=4,211. HRs adjusting for baseline characteristics: age, ethnicity, smoking and marital status.

**Appendix E: Analysis on Healthy Agers**

In order to explore the influence of existing health on the estimated relationships, we ran the analysis on a subset of individuals who were non-smokers and did not have any long-standing illness at baseline. In line with Bowman *et al* (2017), we refer to this sample as ‘healthy agers’ and the sample size is reduced to 3,189. Figure E1 shows the BMI trajectories estimated using this restricted sample. Similar to the main results, we find the same four BMI trajectories, albeit that the elevated BMI trajectory does not show the same persistence and has a much smaller probability than in the full sample. This is not surprising given that this sample is generally healthier. There is also an increased probability of following the increasing BMI trajectory.

Table E1 shows the odds ratios for probability of trajectory membership for healthy agers. The results are not significantly different to the main results in Table 2; confidence intervals overlap with those from the main results.

Table E2 shows the hazard ratios for health outcomes in the healthy agers subsample. It was not possible to estimate all health outcomes form the main analysis because of a lack of model convergence due to large number of parameters estimated alongside the smaller sample. For this reason, hazard ratios for mortality and stroke are not estimated. The confidence intervals for the hazard ratios, particularly in comparing elevated BMI to stable BMI are very wide, also due to the smaller sample size and particularly in the smaller probability of following certain trajectories. Other than that, the results show a similar pattern to the main results in Table 3; all results found to be insignificant in this subsample which were significant in the main sample, have the same sign.

**Figure E1: BMI Trajectories in Healthy Agers Sample**


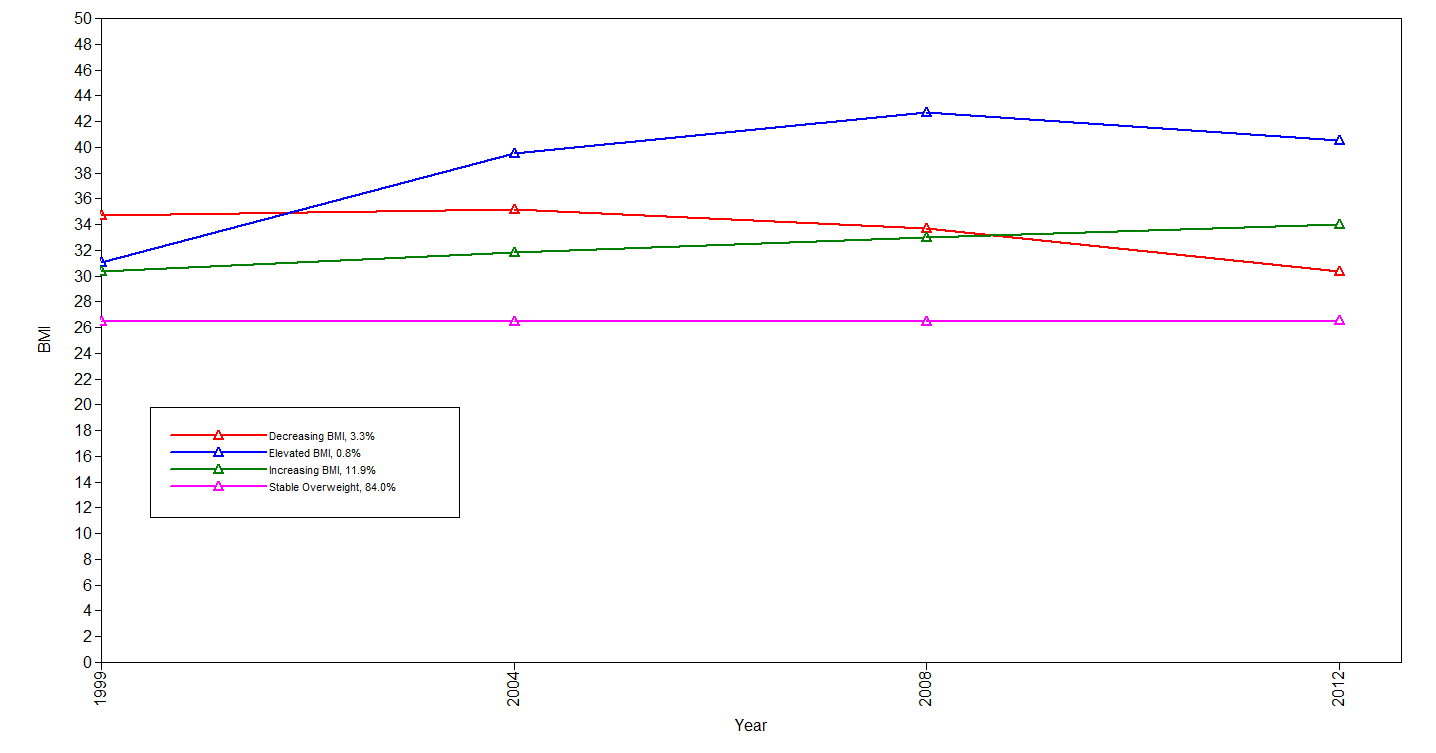


Data source: English Longitudinal Study of Aging (ELSA) and Health Survey for England (HSE), n=3,189

**Table E1: Odds Ratios for Probability of Trajectory in Healthy Agers**

|  | **Stable BMI**  **(reference component)** | **Increasing BMI** | **Elevated BMI** | **Decreasing BMI** |
| --- | --- | --- | --- | --- |
| Mean probability of component membership | 84.0% | 11.9% | 0.8% | 3.3% |
| **Odds Ratios for baseline characteristics (95% Confidence Intervals)** | | | | |
| Age (years) | 1 | **0.951**  **(0.929, 0.974)** | **0.911**  **(0.853, 0.972)** | 1.031  (0.972, 1.092) |
| Male | 1 | **0.353**  **(0.235, 0.532)** | 0.285  (0.081, 1.007) | **0.234**  **(0.112, 0.489)** |
| White | 1 | 1.512  (0.256, 8.935) | 0.299  (0.040, 2.256) | **0.261**  **(0.079, 0.866)** |
| Married | 1 | 0.757  (0.502, 1.141) | 0.557  (0.216, 1.436) | 0.638  (0.335, 1.215) |

Data source: English Longitudinal Study of Aging (ELSA) and Health Survey for England (HSE), n=3,189. Entropy = 0.860.

**Table E2: Hazard Ratios in Healthy Agers**

|  | **Stable BMI**  **(reference trajectory)** | **Increasing BMI** | **Elevated BMI** | **Decreasing BMI** |
| --- | --- | --- | --- | --- |
| Mean probability of trajectory membership | 84.0% | 11.9% | 0.8% | 3.3% |
| **Hazard Ratios (95% Confidence Intervals)** | | | | |
| Mortality | 1 | - | - | **-** |
| Diabetes | 1 | **2.756**  **(1.503, 5.056)** | **9.517**  **(3.890, 20.164)** | **7.430**  **(3.683, 13.393)** |
| Cancer | 1 | 1.063  (0.642, 1.757) | 0.336  (0.014, 8.150) | 1.709  (0.887, 3.294) |
| Arthritis | 1 | **1.814**  **(1.371, 2.401)** | 1.622  (0.737, 3.566) | **2.081**  **(1.314, 3.296)** |
| Asthma | 1 | 1.579  (0.886, 2.814) | 2.275  (0.617, 8.387) | **2.303**  **(1.017, 5.215)** |
| Stroke | 1 | - | - | - |
| Heart Problems | 1 | 1.404  (0.934, 2.109) | 0.919  (0.207, 4.083) | 1.674  (0.972, 2.883) |

Data source: English Longitudinal Study of Aging (ELSA) and Health Survey for England (HSE), n=3,189. HRs adjusting for baseline characteristics: sex, age, ethnicity and marital status.

**Appendix F: Complete case analysis**

In order to explore the influence of missing data on the estimated relationships, we ran the analysis on a subset of individuals who had complete BMI data, resulting in a reduced sample size of 2,979. Figure F1 shows the BMI trajectories estimated using this restricted sample. Similar to the main results, we find the same four BMI trajectories, and like the previous subsample of healthy agers, elevated BMI trajectory does not show the same persistence and has a much smaller probability than in the full sample. This sample is also likely to be healthier than the full sample, given the reduced rate of attrition in heather individuals. The reduced probability of following the elevated BMI trajectory could be due to individuals with missing BMI values due to death in later waves. There is also an increased probability of following the increasing BMI trajectory most likely because this sample will be younger on average.

Table F1 shows the odds ratios for probability of trajectory membership for complete cases. Due to the lower probability of following the elevated BMI trajectory, it was not possible to estimate all odds ratios in the group and the parameter estimate for ethnicity in the elevated BMI group was fixed by Mplus to allow convergence. The results are not significantly different to the main results in Table 2; confidence intervals overlap with those from the main results.

Table F2 shows the hazard ratios for health outcomes in the complete case subsample. The results show a similar pattern to the main results in Table 3. No estimates are available for the mortality hazard ratio because in order to provide complete BMI data across all waves, participants were required to be alive. The elevated BMI trajectory has some wide confidence intervals for the hazard ratios, influencing the significance of any results found. Similarly, there is a smaller probability of following the decreasing BMI in this sample, again, this could influence the significance of results, but overall the parameter estimates remain similar for all trajectories.

**Figure F1: BMI Trajectories in Complete Cases**


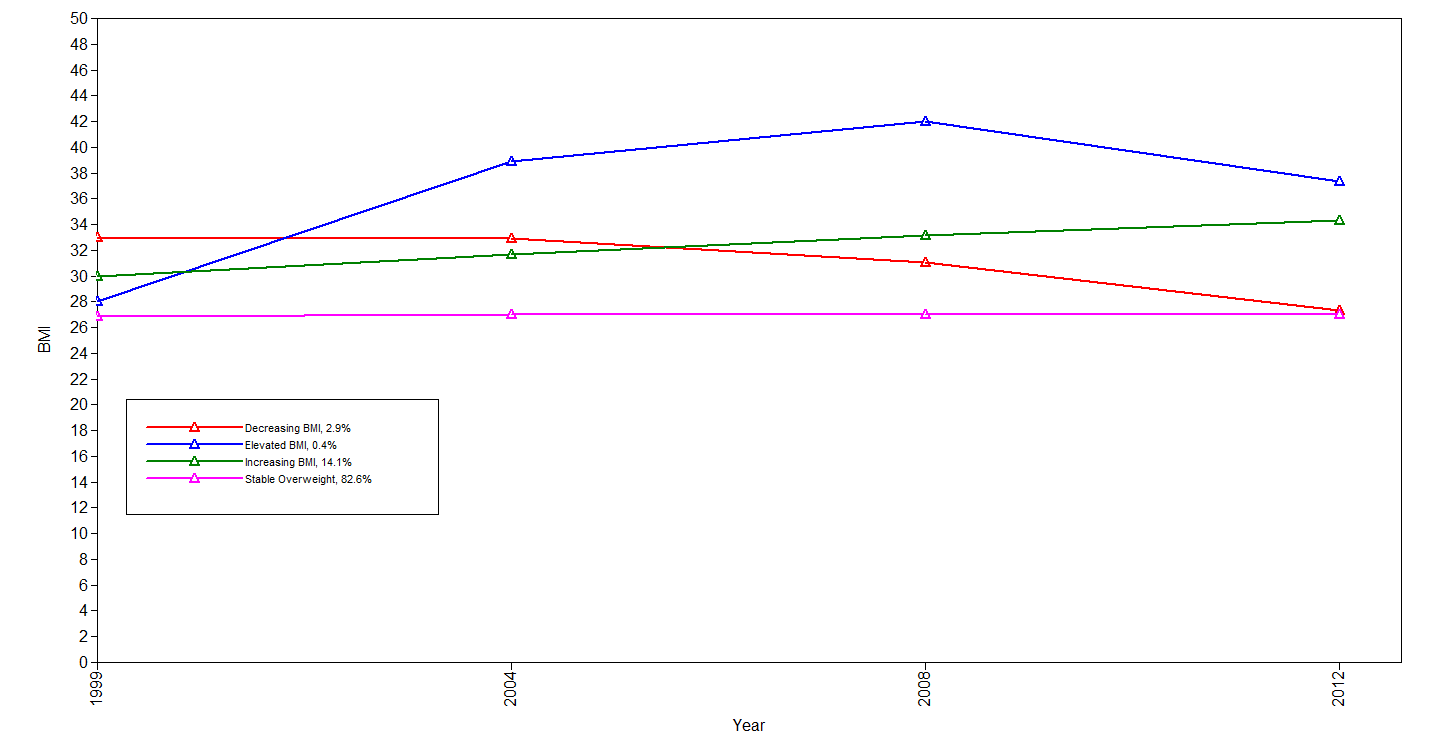
 Data source: English Longitudinal Study of Aging (ELSA) and Health Survey for England (HSE), n=2,979

**Table F1: Odds Ratios for Probability of Trajectory Membership for Complete Cases**

|  | **Stable BMI**  **(reference component)** | **Increasing BMI** | **Elevated BMI** | **Decreasing BMI** |
| --- | --- | --- | --- | --- |
| Mean probability of component membership | 82.6% | 14.1% | 0.4% | 2.9 % |
| **Odds Ratios for baseline characteristics (95% Confidence Intervals)** | | | | |
| Age (years) | 1 | **0.945**  **(0.924, 0.968)** | 0.888  (0.774, 1.019) | **1.071**  **(1.024, 1.121)** |
| Male | 1 | **0.382**  **(0.273, 0.535)** | 0.508  (0.153, 1.686) | **0.341**  **(0.163, 0.713)** |
| White | 1 | 1.113  (0.275, 4.507) | - | 0.599  (0.091, 3.924) |
| Smoker | 1 | **2.756**  **(1.919, 3.957)** | 2.707  (0.757, 9.684) | 1.682  (0.552, 5.121) |
| Married | 1 | 0.823  (0.570, 1.189) | 1.075  (0.306, 3.778) | 0.616  (0.286, 1.326) |

Data source: English Longitudinal Study of Aging (ELSA) and Health Survey for England (HSE), n=2,979. Entropy = 0.883.

**Table F2: Hazard Ratios for Morbidities and Mortality for Complete Cases**

|  | **Stable BMI**  **(reference trajectory)** | **Increasing BMI** | **Elevated BMI** | **Decreasing BMI** |
| --- | --- | --- | --- | --- |
| Mean probability of trajectory membership | 82.6% | 14.1% | 0.4% | 2.9 % |
| **Hazard Ratios (95% Confidence Intervals)** | | | | |
| Mortality | 1 | - | - | **-** |
| Diabetes | 1 | **2.310**  **(1.479, 3.607)** | **6.795**  **(2.642, 17.474)** | **3.567**  **(1.908, 6.669)** |
| Cancer | 1 | 1.022  (0.636, 1.643) | 1.512  (0.355, 6.442) | 1.508  (0.784, 2.900) |
| Arthritis | 1 | **1.636**  **(1.243, 2.154)** | 1.648  (0.566, 4.798) | 1.671  (0.987, 2.828) |
| Asthma | 1 | **1.868**  **(1.104, 3.159)** | 1.537  (0.199, 11.897) | 1.485  (0.540, 4.082) |
| Stroke | 1 | 1.073  (0.513, 2.242) | 2.087  (0.265, 16.463) | 0.974  (0.367, 2.586) |
| Heart Problems | 1 | 1.208  (0.840, 1.737) | 2.096  (0.762, 5.766) | 1.321  (0.803, 2.174) |

Data source: English Longitudinal Study of Aging (ELSA) and Health Survey for England (HSE), n=2,979. HRs adjusting for baseline characteristics: sex, age, ethnicity, smoking and marital status.

**Appendix G: Exclusion of Underweight**

In order to determine whether underweight individuals could be biasing the results, particularly given that we do not identify an underweight trajectory, we estimate the model with participants removed if they reported being underweight (BMI<18) in any wave. Very few observations (103) fell into this criteria and the resulting sample size was 9,103.

Figure G1 shows the BMI trajectories estimated using this sample; they remain very similar to the main results in Figure 1. Similarly, Tables G1 and G2 show very similar results to the main sample. Underweight individuals do not appear to be influencing the results.

**Figure G1: BMI Trajectories in Sample excluding Underweight**


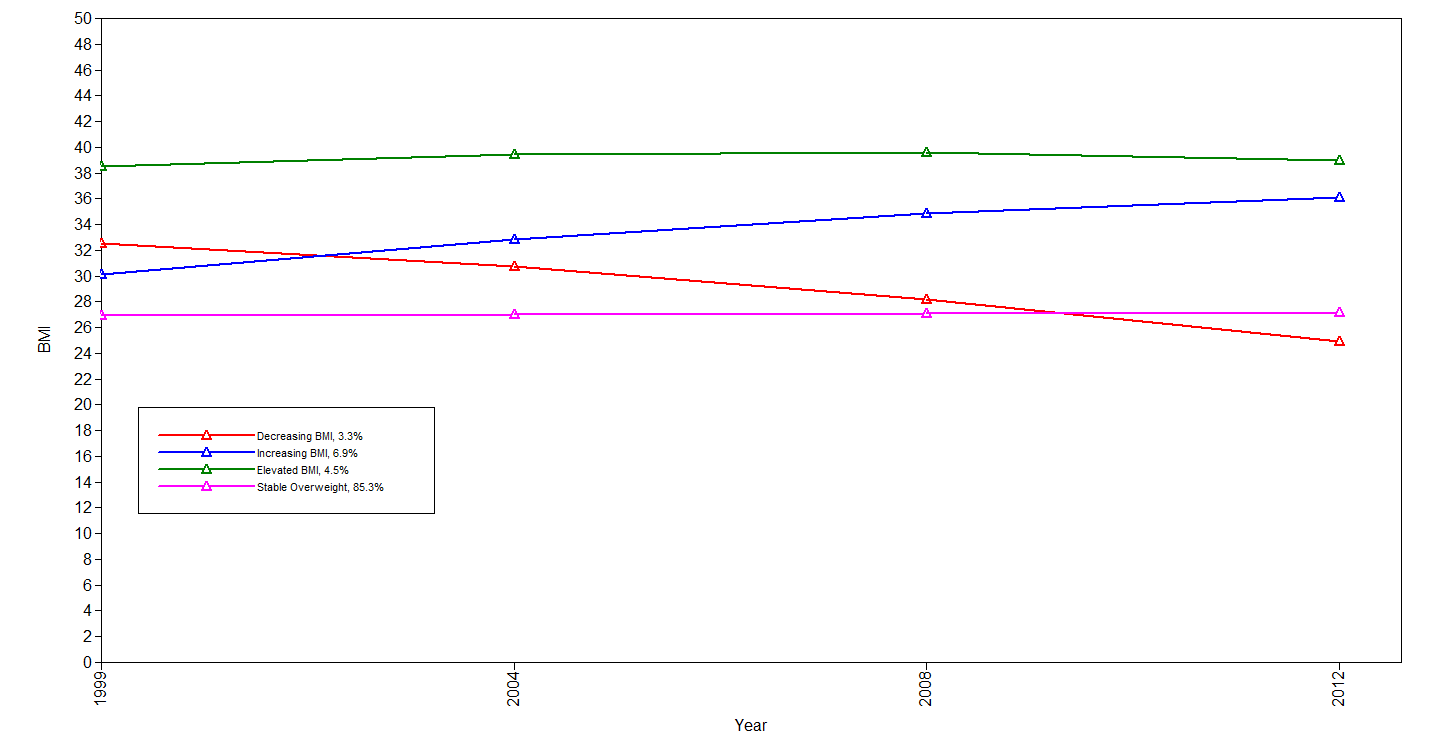


Data source: English Longitudinal Study of Aging (ELSA) and Health Survey for England (HSE), n=9,103

**Table G1: Odds Ratios for Probability of Trajectory Membership excluding Underweight**

|  | **Stable BMI**  **(reference component)** | **Increasing BMI** | **Elevated BMI** | **Decreasing BMI** |
| --- | --- | --- | --- | --- |
| Mean probability of component membership | 85.3% | 6.9% | 4.5% | 3.3% |
| **Odds Ratios for baseline characteristics (95% Confidence Intervals)** | | | | |
| Age (years) | 1 | **0.945**  **(0.928, 0.962)** | **0.971**  **(0.955, 0.988)** | **1.088**  **(1.051, 1.127)** |
| Male | 1 | **0.448**  **(0.347, 0.577)** | **0.267**  **(0.185, 0.387)** | **0.203**  **(0.108, 0.397)** |
| White | 1 | 1.316  (0.505, 3.433) | 0.477  (0.183, 1.248) | 1.143  (0.092, 14.170) |
| Smoker | 1 | **2.500**  **(1.917, 3.259)** | 0.725  (0.468, 1.122) | **1.707**  **(1.015, 2.872)** |
| Married | 1 | 0.883  (0.688, 1.133) | **0.584**  **(0.418, 0.816)** | 0.688  (0.461, 1.026) |

Data source: English Longitudinal Study of Aging (ELSA) and Health Survey for England (HSE), n=9,103. Entropy = 0.871.

**Table G2: Hazard Ratios for Morbidities and Mortality excluding Underweight**

|  | **Stable BMI**  **(reference trajectory)** | **Increasing BMI** | **Elevated BMI** | **Decreasing BMI** |
| --- | --- | --- | --- | --- |
| Mean probability of trajectory membership | 85.3% | 6.9% | 4.5% | 3.3% |
| **Hazard Ratios (95% Confidence Intervals)** | | | | |
| Mortality | 1 | 0.862  (0.460, 1.613) | 1.003  (0.555, 1.814) | 1.197  (0.840, 1.705) |
| Diabetes | 1 | **3.679**  **(2.693, 5.027)** | **5.644**  **(4.070, 7.827)** | **4.432**  **(2.830, 6.943)** |
| Cancer | 1 | 0.875  (0.562, 1.362) | 1.263  (0.824, 1.938) | 1.053  (0.611, 1.816) |
| Arthritis | 1 | **1.762**  **(1.375, 2.260)** | **1.661**  **(1.206, 2.289)** | 1.052  (0.660, 1.676) |
| Asthma | 1 | **1.828**  **(1.183, 2.824)** | **2.120**  **(1.287, 3.491)** | **2.674**  **(1.419, 5.040)** |
| Stroke | 1 | 1.411  (0.802, 2.481) | 1.017  (0.498, 2.077) | 1.146  (0.627, 2.097) |
| Heart Problems | 1 | 1.258  (0.927, 1.708) | **1.571**  **(1.142, 2.162)** | 1.322  (0.892, 1.959) |

Data source: English Longitudinal Study of Aging (ELSA) and Health Survey for England (HSE), n=9,103. HRs adjusting for baseline characteristics: sex, age, ethnicity, smoking and marital status.

**Appendix H: Inclusion of IMD quintile**

In order to determine whether socio-economic status had an influence on our results, we performed a secondary analysis adjusting for IMD quintile it in both Step 1 and Step 2 of our analysis. This variable was not included in the main analysis because it reduced the sample size substantially and its inclusion made little difference to our results. We assume IMD quintile to be time invariant.

Figure H1 shows the BMI trajectories adjusted for IMD quintile; they remain very similar to the main results in Figure 1. Similarly, Tables H1 and H2 show very similar results to the main results. The inclusion of IMD quintile does not appear to be substantially influencing the results. There is some evidence of a significant effect of IMD quintile increasing the odds of following the decreasing BMI trajectory compared to the stable overweight category, but this is small and this results is taken from a reduced sample. In some cases, HRs are insignificant once adjusting for IMD quintile (arthritis and heart problems in the elevated BMI group and asthma in the decreasing BMI group), however, the coefficients remain similar, and they move from only just significant (95%), to only just insignificant. We see no substantial difference in the results.

**Figure H1: BMI Trajectories adjusting for IMD quintile**


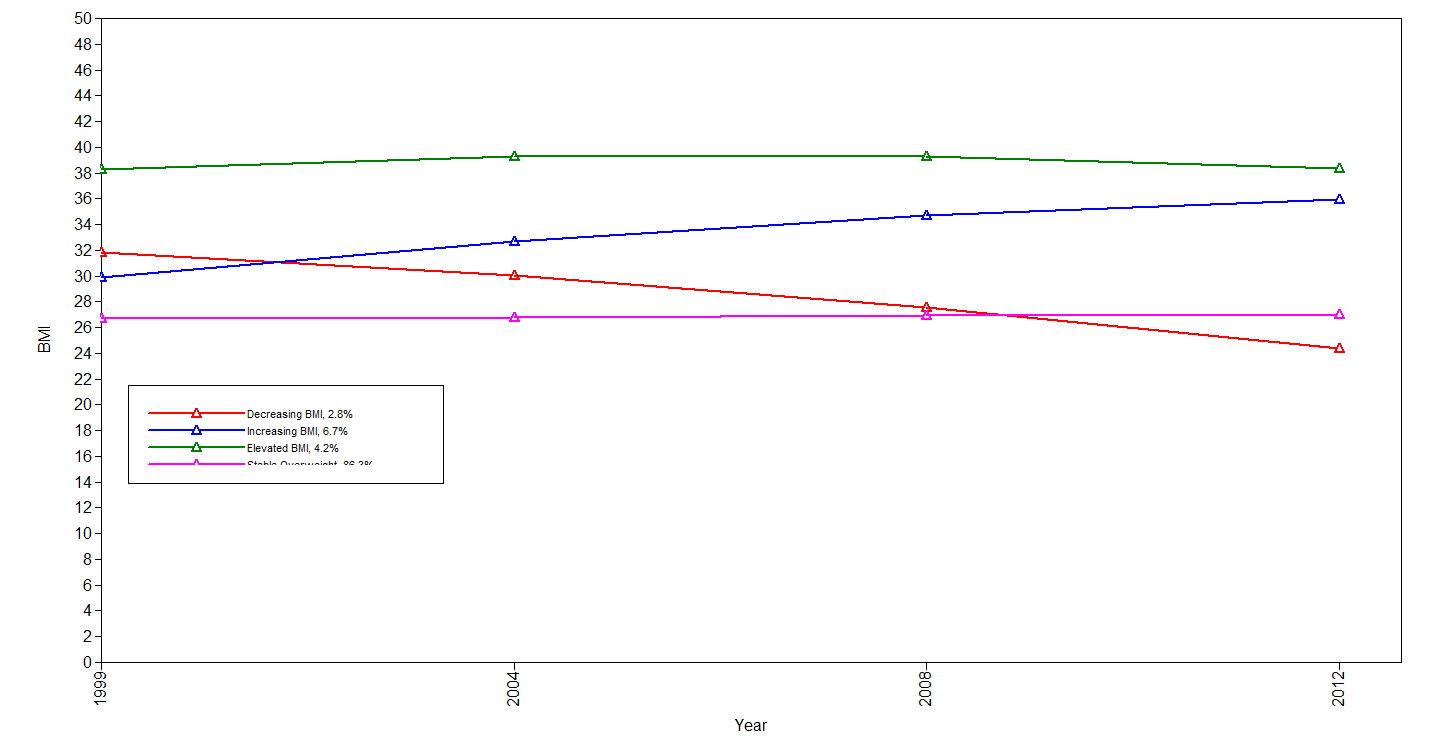


Data source: English Longitudinal Study of Aging (ELSA) and Health Survey for England (HSE), n=7,461

**Table H1: Odds Ratios for Probability of Trajectory Membership**

|  | **Stable BMI**  **(reference component)** | **Increasing BMI** | **Elevated BMI** | **Decreasing BMI** |
| --- | --- | --- | --- | --- |
| Mean probability of component membership | 86.3% | 6.7% | 4.2% | 2.8% |
| **Odds Ratios for baseline characteristics (95% Confidence Intervals)** | | | | |
| Age (years) | 1 | **0.947**  **(0.927, 0.967)** | **0.973**  **(0.951, 0.995)** | **1.110**  **(1.059, 1.164)** |
| Male | 1 | **0.395**  **(0.292, 0.534**) | **0.277**  **(0.183, 0.422)** | **0.151**  **(0.061, 0.375)** |
| White | 1 | 1.820  (0.620, 5.338) | 0.657  (0.279, 1.549) | 1.149  (0.107, 12.395) |
| Smoker | 1 | **2.506**  **(1.854, 3.386)** | 0.695  (0.394, 1.225) | 1.835  (0.952, 3.535) |
| Married | 1 | 0.920  (0.699, 1.210) | **0.666**  **(0.451, 0.984)** | **1.030**  **(1.130, 1.532)** |
| IMD quintile | 1 | 1.179  (0.971, 1.397) | 1.240  (0.950, 1.565) | **1.316**  **(1.030, 1.632)** |

Data source: English Longitudinal Study of Aging (ELSA) and Health Survey for England (HSE), n=7,461. Entropy = 0.882.

**Table H2: Hazard Ratios for Morbidities and Mortality adjusting for IMD quintile**

|  | **Stable overweight**  **(reference trajectory)** | **Increasing BMI** | **Elevated BMI** | **Decreasing BMI** |
| --- | --- | --- | --- | --- |
| Mean probability of trajectory membership | 86.3% | 6.7% | 4.2% | 2.8% |
| **Hazard Ratios (95% Confidence Intervals)** | | | | |
| Mortality | 1 | 0.735  (0.349, 1.548) | 1.248  (0.630, 2.469) | 1.152  (0.751,1.768) |
| Diabetes | 1 | **4.793**  **(3.266, 7.034)** | **6.072**  **(3.977, 9.269)** | **4.557**  **(2.363, 8.786)** |
| Cancer | 1 | 0.948  (0.588, 1.528) | 1.239  (0.740, 2.073) | 1.450  (0.804, 2.615) |
| Arthritis | 1 | **1.662**  **(1.279, 2.159)** | 1.353  (0.899, 2.038) | 1.212  (0.720, 2.042) |
| Asthma | 1 | **1.673**  **(1.040, 2.689)** | **2.174**  **(1.195, 3.955)** | 2.198  (0.947, 5.106) |
| Stroke | 1 | 1.408  (0.684, 2.898) | 1.539  (0.682, 3.472) | 1.984  (0.947, 4.155) |
| Heart Problems | 1 | 1.250  (0.894, 1.748) | 1.378  (0.930, 2.043) | 1.220  (0.743, 2.001) |

Data source: English Longitudinal Study of Aging (ELSA) and Health Survey for England (HSE), n=7,461. HRs adjusting for baseline characteristics: sex, age, smoking status, ethnicity, marital status and IMD quintile.
